# Gamma synuclein is a novel Twist1 target that promotes TGF-β-induced cancer cell migration and invasion

**DOI:** 10.1038/s41419-018-0657-z

**Published:** 2018-05-24

**Authors:** Ting Shao, Peiying Song, Hui Hua, Hongying Zhang, Xiangmin Sun, Qingbin Kong, Jiao Wang, Ting Luo, Yangfu Jiang

**Affiliations:** 1 State Key Laboratory of Biotherapy, Section of Oncogene, Cancer Center, West China Hospital, Sichuan University and Collaboratory Inovation Center for Biotherapy, Chengdu, China; 2 Laboratory of Stem Cell Biology, West China Hospital, Sichuan University, Chengdu, China; 3 School of Basic Medicine, Chengdu University of Traditional Chinese Medicine, Chengdu, China; 4 Cancer Center, West China Hospital, Sichuan University, Chengdu, China

## Abstract

Transforming growth factor β (TGF-β) is critical for embryonic development, adult tissue homeostasis, and tumor progression. TGF-β suppresses tumors at early stage, but promotes metastasis at later stage through oncogenes such as Twist1. Gamma-synuclein (SNCG) is overexpressed in a variety of invasive and metastatic cancer. Here, we show that TGF-β induces SNCG expression by Smad-Twist1 axis, thus promoting TGF-β- and Twist1-induced cancer cell migration and invasion. We identify multiple Twist1-binding sites (E-boxes) in *SNCG* promoter. Chromatin immunoprecipitation and luciferase assays confirm the binding of Twist1 to the E-boxes of *SNCG* promoter sequence (−129/−1026 bp). Importantly, the Twist1-binding site close to the transcription initiation site is critical for the upregulation of SNCG expression by TGF-β and Twist1. Mutations of Twist1 motif on the *SNCG* promoter constructs markedly reduces the promoter activity. We further show that TGF-β induces Twist1 expression through Smad thereby enhancing the binding of Twist1 to SNCG promoter, upregulating *SNCG* promoter activity and increasing SNCG expression. SNCG knockdown abrogates TGF-β- or Twist1-induced cancer cell migration and invasion. Finally, SNCG knockdown inhibits the promotion of cancer metastasis by Twist1. Together, our data demonstrate that SNCG is a novel target of TGF-β-Smad-Twist1 axis and a mediator of Twist1-induced cancer metastasis.

## Introduction

Gamma-synuclein (SNCG) is one of the three members of the synuclein family (α-synuclein/SNCA, β-synuclein/SNCB, and SNCG), which are preferentially expressed in the brain and peripheral nervous system. SNCA is found mainly at presynaptic terminals where it plays a role in clustering synaptic vesicles and promoting SNARE-complex assembly thereby regulating the release of neurotransmitters^1,2^. While the biophysical properties of native SNCA remains controversial^3,4^, SNCA is susceptible to aggregation, which is involved in Alzheimer’s disease, Parkinson disease, dementia with Lewy bodies, and multiple system atrophy^5,6^. SNCB and SNCG, however, have antagonistic effects on SNCA aggregation^7^. Normally, SNCG is expressed in peripheral neurons, ocular tissue, and adipose^8,9^. In addition, SNCG is overexpressed in various types of human tumors, such as breast, ovary, colon, liver, and cervical cancer^10–13^. Overexpression of SNCG in cancer cells may be due to aberrant demethylation of CpG islands within the promoter, AP1 transactivation, and insulin-like growth factor signaling^13–15^. SNCG promotes cancer metastasis and cancer cell survival under stresses^16–19^. Upon interacting with heat-shock proteins (HSPs), SNCG acts as a co-chaperone of HSP to stimulate estrogen receptor signaling^20^. The stability or activity of multiple kinases, such as IGF-1R, Akt, and ERK1/2, is enhanced by SNCG^14,19,21^. Moreover, SNCG interacts with BubR1 to regulate cell cycle checkpoint^22^. Therefore, SNCG may promote tumor progression and drug resistance through multiple mechanisms. Overexpression of SNCG is a predictive marker for poor prognosis in human breast cancer^11^.

Similar to SNCG, the basic helix-loop-helix transcription factor Twist1 acts as an oncogene in many cancers including breast cancer, hepatocellular carcinoma, pancreatic carcinoma, and neuroblastoma^23–25^. Twist1 can be activated by a variety of signal transduction pathways, including signal transducer and activator of transcription 3 (STAT3), Ras, mitogen-activated protein kinase (MAPK), and Wnt signaling^26,27^. Twist1 preferentially binds to E-box (5′-CANNTG-3′) consensus sites in the promoter of target genes and regulate gene expression^28^. Activated Twist1 upregulates N-cadherin and downregulates E-cadherin, which are the hallmarks of epithelial–mesenchymal transition (EMT), a process characterized by loss of cell–cell contacts and acquisition of fibroblastic phenotypes^29^. EMT is important for embryonic development, cancer metastasis, and drug resistance^30,31^. In addition, Twist1 upregulates the expression of matrix metalloproteinases, which degrades the extracellular matrix (ECM) and paves the way for cell dissemination^27^. Moreover, Twist1 promotes cancer metastasis by regulating multiple processes involved in metastasis, such as angiogenesis, invasion, migration, extravasation, and chromosomal instability^32,33^. Twist1 is responsible for the maintenance of cancer stem cells and the development of chemotherapy resistance^34–36^.

Twist1 expression can be induced by transforming growth factor-β (TGF-β), a pleiotrophic cytokine that may inhibit cell proliferation, promote cell differentiation, invasion, migration, and immune evasion^37,38^. While TGF-β inhibits tumorigenesis at the early stage, it often promotes tumor progression at the late stage. During tumor progression, TGF-β frequently switchs it’s function from growth arrest to promotion of cancer cell survival, EMT, migration, invasion, vascularization, metastasis, and immunosuppression^38^. Hence, the immunosuppresive and pro-metastasis functions of TGF-β may come to dominate in late-stage cancer. While the CDK inhibitors p21Cip1 and p15Ink4b mediate the inhibition of cell proliferation by TGF-β, the transcription factors such as Snail are induced by TGF-β to promote EMT^39^. While EMT is generally considered a pro-tumor event, recent study also demonstrates that TGF-β suppresses pancreatic ductal adenocarcinoma through a lethal EMT^40^.

Thus far, it is unknown whether SNCG is involved in TGF-β-induced cell invasion and migration. Here, we report that SNCG is a TGF-β responsive protein. TGF-β induces SNCG expression through Twist1, which bind to E-boxes in the promoter of *SNCG* thereby stimulating *SNCG* transcription. SNCG promotes TGF-β- and Twist1-induced cancer cell invasion and migration. SNCG knockdown inhibits the promotion of cancer metastasis by Twist1.

## Results

### Twist1 upregulates SNCG expression

**Fig. 1:**
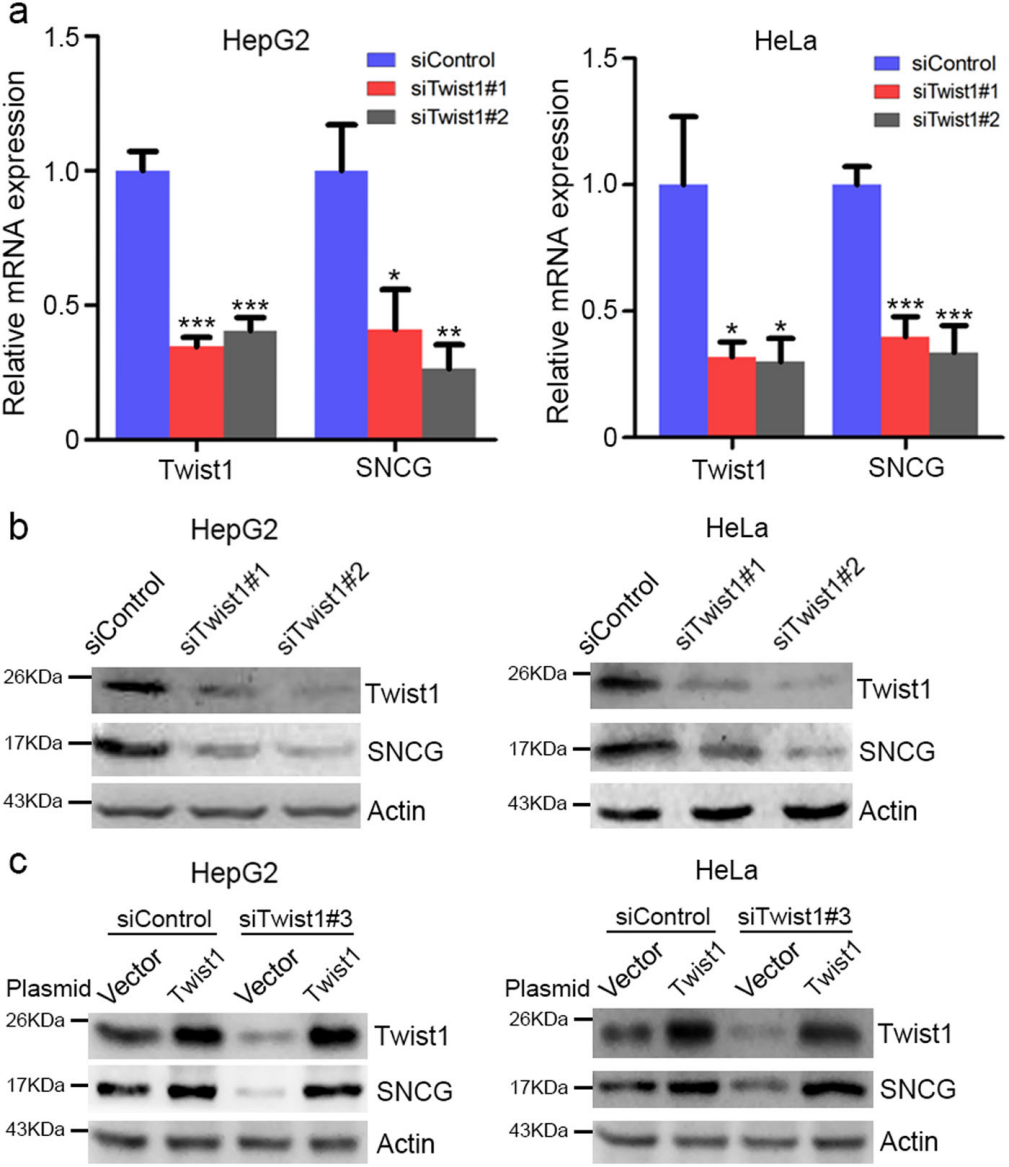
Twist1 upregulates SNCG expression. **a** HepG2 and HeLa cells were transfected with 50 nM of the indicated siRNA. Twenty-four hours after transfection, qRT-PCR was conducted using the primers for *Twist1*, *SNCG*, and *GAPDH*. The relative mRNA expression was plotted. The level of transcripts in cells transfected with control siRNA (siControl) was set as 1. The values represent mean ± S.D. (*n* = 3). *p < 0.05; **p < 0.01; ***p < 0.001, compared with siControl. **b** HepG2 and HeLa cells were transfected with 50 nM of the indicated siRNA. Forty-eight hours after transfection, whole-cell extracts were analyzed by western blot analysis with indicated antibodies. **c** HepG2 and HeLa cells were transfected with 50 nM of the indicated siRNA. Twenty-four hours after transfection, the cells were transfected with 1 μg/ml of the indicated plasmid. Forty-eight hours after transfection, whole-cell extracts were analyzed by western blot analysis with indicated antibodies

To decipher the regulatory mechanism of SNCG expression in cancer cells, we analyzed human *SNCG* promoter. There are multiple consensus E-boxes in human *SNCG* promoter (Supplementary Fig. 1). Twist1 is an E protein that binds to E-box thereby regulating gene expression. To determine whether Twist1 regulates SNCG expression, HepG2 cells were transfected with or without Twist1 small interfering RNA (siRNA) (siTwist1#1 or siTwist1#2), followed by real-time reverse transcription (RT)-PCR analysis of *SNCG* and *Twist1* transcription. Knockdown of Twist1 by two sets of siRNA consistently led to a decrease in *SNCG* transcription (Fig. 1a). Moreover, Twist1 knockdown inhibited SNCG expression at protein level (Fig. 1b). Similar effects were detected in HeLa cells (Fig. 1a, b). Knockdown of Twist1 by the third siRNA (siTwist1#3) targeting 3′-untranslated region of *Twist1* also inhibited SNCG expression (Fig. 1c). Overexpression of siTwist1#3-resistant Twist1 upregulated SNCG expression and rescued the decrease in SNCG expression resulting from Twist1 knockdown (Fig. 1c). Taken together, these data demonstrate that Twist1 positively regulates SNCG expression.

### Twist1 upregulates SNCG transcription through E-boxes in SNCG promoter

**Fig. 2:**
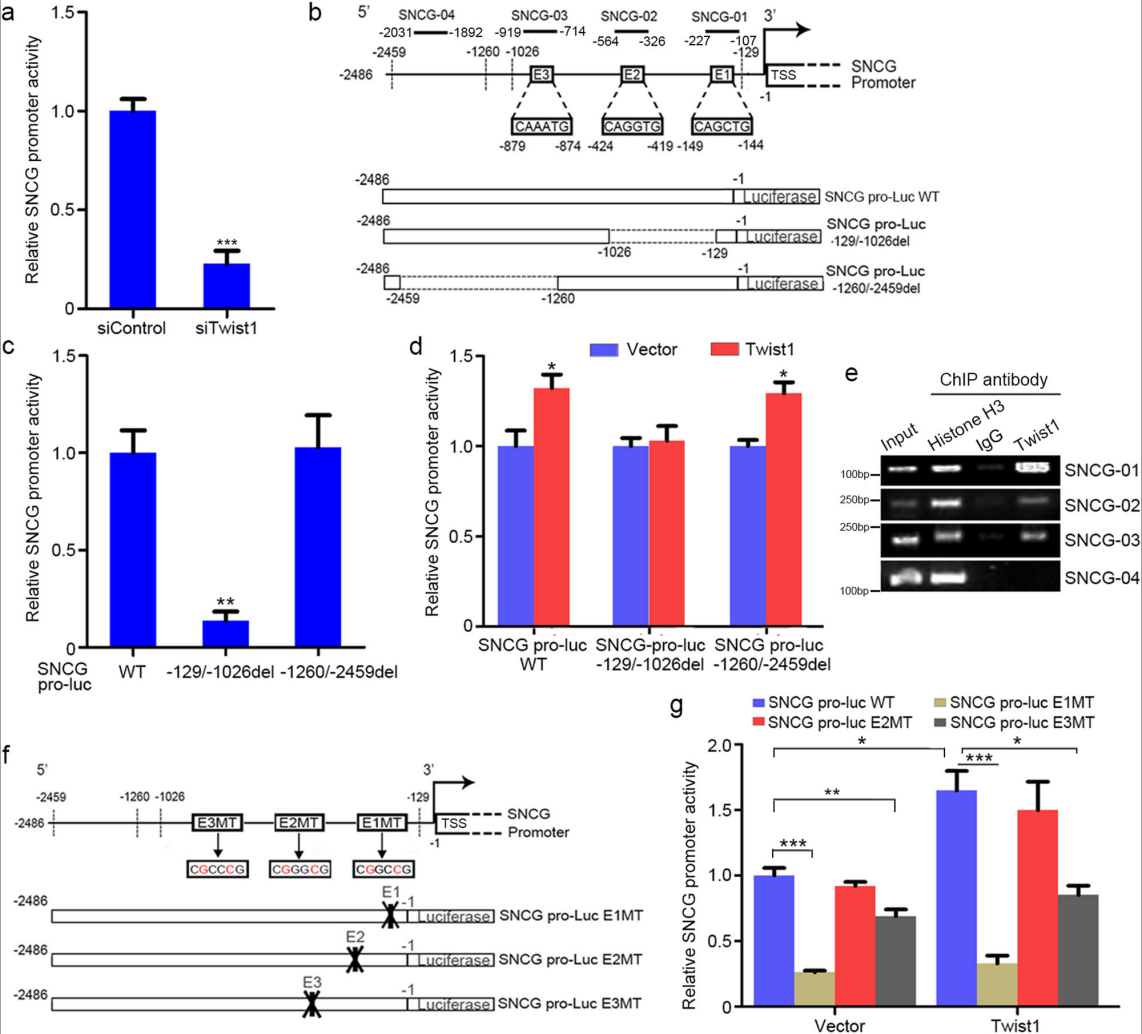
Twist1 upregulates *SNCG* transcription by binding to E-boxes in *SNCG* promoter. **a** HepG2 cells were transfected with 50 nM of the indicated siRNA. Twenty-four hours after transfection, cells were transfected with 0.5 μg/ml of the SNCG pro-Luc plasmid, followed by detection of luciferase activities after another 24 h. The luciferase activity in siControl-transfected cells was set as 1. Values represent mean ± S.D. (*n* = 4); ***p < 0.001, compared with siControl. **b** Schematic representation of the promoter region (−1/−2486) of the SNCG gene. The three E-boxes (E1, E2, and E3) within −129/−1026 bp were shown. The SNCG promoter reporter constructs were also shown. **c** HepG2 cells were transfected with 0.5 μg/ml of the various SNCG pro-Luc plasmids shown in (**b**), followed by detection of luciferase activities. The luciferase activity of SNCG pro-Luc WT was set as 1. Values represent mean ± S.D. (*n* = 3). **p < 0.01, compared with SNCG pro-Luc WT. **d** HepG2 cells were transfected with Twist1 overexpression plasmid. Twenty-four hours after transfection, the cells were transfected with 0.5 μg/ml of the various SNCG pro-Luc plasmids shown in (**b**), followed by detection of luciferase activities after another 24 h. The luciferase activity in vector-transfected cells was set as 1. Values represent mean ± S.D. (*n* = 3). *p < 0.05, compared with vector-transfected cells. **e** HepG2 cells were lysed and sonicated into 200–1000 bp fragments after fixed with formaldehyde. Soluble chromatin was co-immunoprecipitated with antibody against Twist1, H3, or IgG to collect Twist1- or H3-binding DNA fragments. The co-immunoprecipitated DNA was subjected to PCR with primers for different regions within SNCG promoter (SNCG-01, SNCG-02, SNCG-03, and SNCG-04), as shown in (**b**). PCR products were subjected to agarose gel electrophoresis. **f** Schematic representation of SNCG promoter-luciferase constructs with mutations in E-boxes (E1MT, E2MT, and E3MT). **g** HepG2 cells were transfected with vector or Twist1 plasmid. Twenty-four hours after transfection, the cells were transfected with 0.5 μg/ml of the various SNCG pro-Luc plasmids shown in (**f**), followed by detection of luciferase activities after another 24 h. The luciferase activity in cells transfected with SNCG pro-Luc WT and vector was set as 1. Values represent mean ± S.D. (*n* = 3). *p < 0.05; **p < 0.01; ***p < 0.001

To elucidate the mechanisms underlying the upregulation of *SNCG* transcription by Twist1, we analyzed the effects of Twist1 on the activity of *SNCG* promoter. We prepared a luciferase reporter containing the 5-flanking region of *SNCG* gene (−1/−2486 bp), in which there are 16 consensus E-boxes (Supplementary Fig. 1). The luciferase reporter analysis revealed that Twist1 knockdown resulted in a significant decrease in *SNCG* promoter activity (Fig. 2a). To further investigate how Twist1 regulates *SNCG* transcription, we analyzed two deletion mutants of *SNCG* promoter (−129/−1026del; −1260/−2459del) (Fig. 2b). The promoter activity of −129/−1026del mutant (SNCG pro−129/−1026del) was significantly lower than that of wild-type promoter (SNCG pro-WT), while the promoter activity of −1260/−2459del mutant (SNCG pro−1260/−2459del) was similar to that of wild-type promoter (SNCG pro-WT) (Fig. 2c). Whereas Twist1 overexpression stimulated the promoter activity of SNCG pro-WT and SNCG pro−1260/−2459del to similar extent, it failed to stimulate the promoter activity of SNCG pro−129/−1026del (Fig. 2d), suggesting that the −129/−1026 bp region within *SNCG* promoter is critical for the stimulation of *SNCG* transcription by Twist1.

There are three putative E-boxes within −129/−1026 bp region, namely E1, E2, and E3 (Fig. 2b). Chromatin immunoprecipitation (ChIP) assays demonstrated that endogenous Twist1 was preferentially recruited to E1- and E3-containing regions, while it was also recruited to E2-containing region to a lesser extent (Fig. 2e). No binding was detected within a distal region (−1892 to −2031 bp) (Fig. 2e), although there are putative E-boxes within this region (Supplementary Fig. 1). To further confirm the regulation of *SNCG* transcription by Twist1-binding via E-boxes, we analyzed the effects of Twist1 overexpression on E-box-disrupted mutants of SNCG promoter (Fig. 2f). Mutations in E1 and E3 reduced SNCG promoter activity and blunted the stimulation of *SNCG* promoter activity by Twist1, whereas E2 mutation had no effects on *SNCG* promoter activity (Fig. 2g). Compared with E3 mutation, E1 mutation more significantly affected *SNCG* promoter activity (Fig. 2g). Taken together, these data demonstrated that Twist1 directly binds and transactivates *SNCG* promoter, and confirms that SNCG is a downstream target of Twist1. The E-box mostly close to the transcription start site was critical for *SNCG* promoter activity and Twist1-induced *SNCG* transcription.

### TGF-β induces SNCG expression via Smad-Twist1 axis

**Fig. 3:**
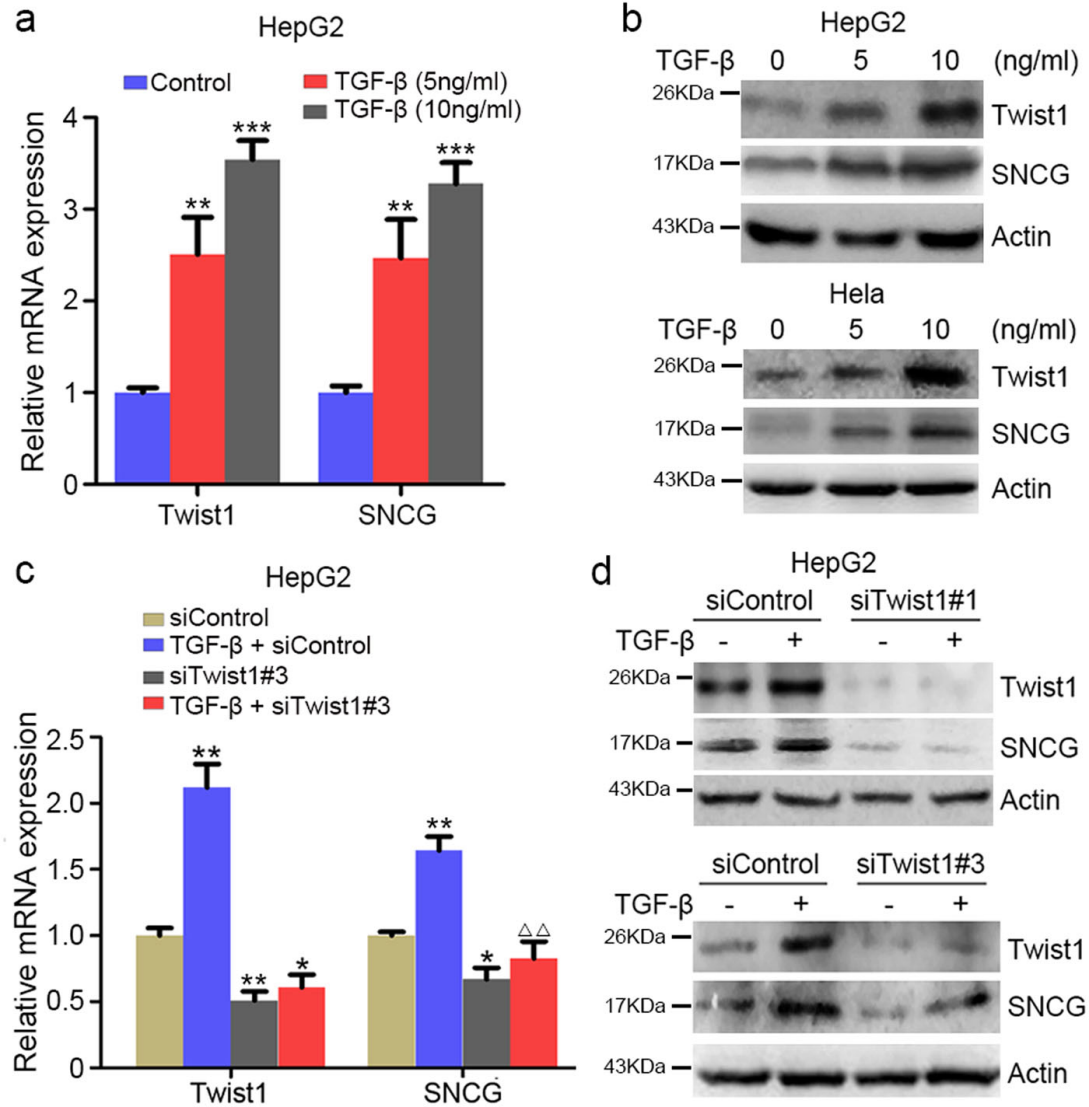
TGF-β induces SNCG expression via Twist1. **a** HepG2 cells were treated with or without TGF-β for 24 h at indicated doses, followed by qRT-PCR using the primers for *Twist1*, *SNCG*, and *GAPDH*. The levels of transcripts in vehicle-treated cells was set as 1. Values represent mean ± S.D. (*n* = 3). **p < 0.01; ***p < 0.001, compared with control. **b** HepG2 and HeLa cells were treated with TGF-β for 48 h at indicated doses. Whole-cell extracts were analyzed by western blotting with indicated antibodies. **c** HepG2 cells were transfected with control siRNA or siTwist1. Twenty-four hours later, the cells were treated with 5 ng/ml TGF-β for another 24 h, followed by qRT-PCR analysis of Twist1, SNCG, and GAPDH. The levels of transcripts in siControl-transfected cells were set as 1. Values represent mean ± S.D. (*n* = 3). *p < 0.05, **p < 0.01, compared with siControl; ^ΔΔ^*p* < 0.01, compared with cells treated with TGF-β. **d** HepG2 cells were transfected with control siRNA or siTwist1#1, siTwist1#3. Twenty-four hours later, the cells were treated with 5 ng/ml of TGF-β for another 48 h. Whole-cell extracts were analyzed by western blotting with indicated antibodies

Since Twist1 is an oncogene downstream of TGF-β signaling, we then detected whether TGF-β could induce SNCG expression at both transcript and protein levels. Treatment of HepG2 cells with TGF-β led to   increased Twist1 and SNCG transcription in a dose-dependent manner (Fig. 3a). Similar effects were detected in HeLa cells (Supplementary Fig. 2a). Also, TGF-β-induced Twist1 and SNCG expression at protein level (Fig. 3b). Moreover, Twist1 knockdown abrogated the induction of SNCG expression by TGF-β in both HepG2 and HeLa cells (Fig. 3c, d; Supplementary Figs. 2b, 3), indicating that TGF-β induces SNCG expression through Twist1.

**Fig. 4:**
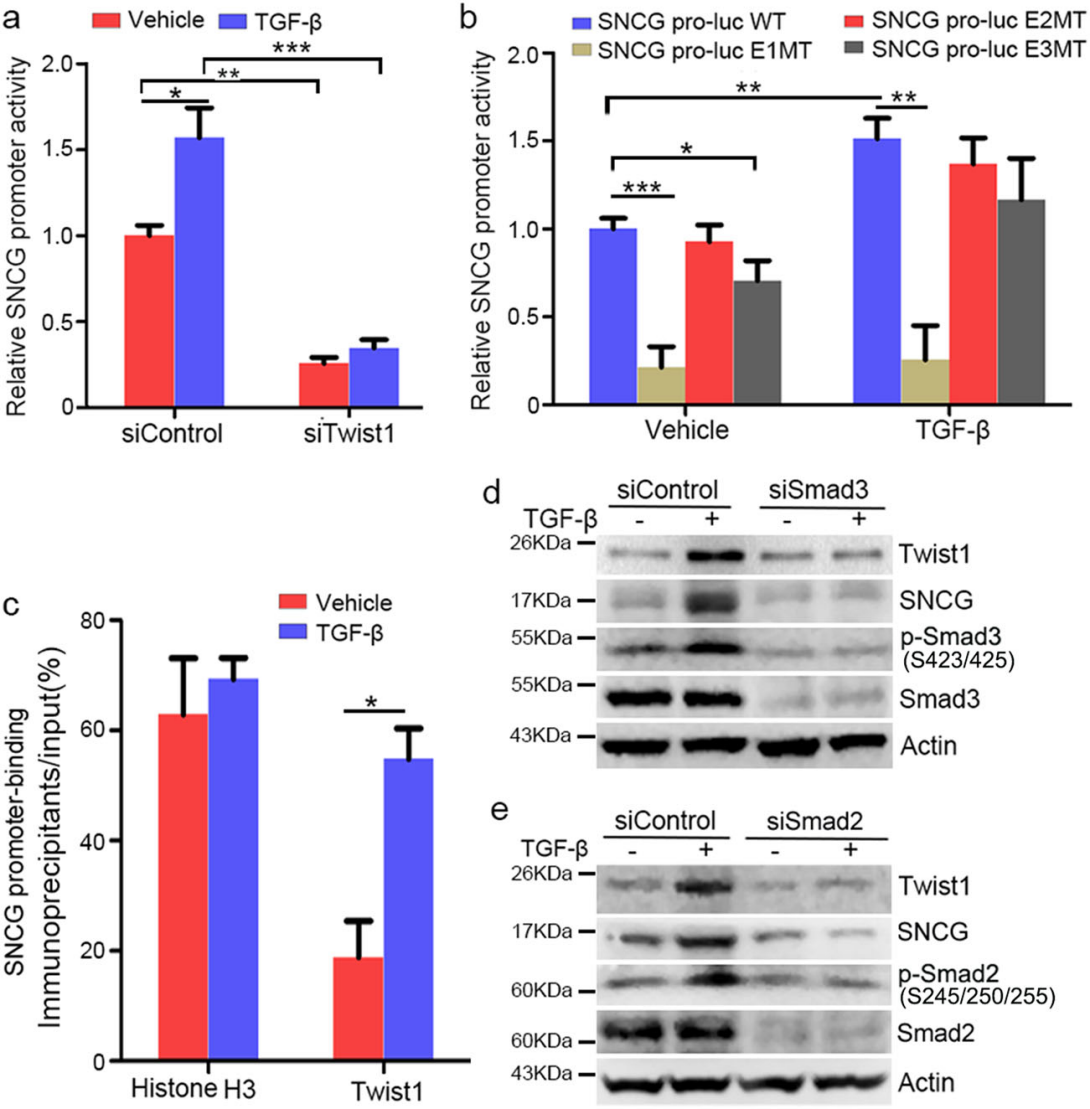
TGF-β stimulates SNCG promoter activity and induces SNCG expression through Smad-Twist1 axis. **a** HepG2 cells were co-transfected with 0.5 μg/ml of SNCG pro-Luc WT plasmid, and 50 nM of indicated siRNA. Twenty-four hours later, the cells were treated with or without 5 ng/ml of TGF-β for another 24 h, followed by detection of luciferase activity. The luciferase activity in cells transfected with SNCG pro-Luc WT and siControl was set as 1. Values represent mean ± S.D. (*n* = 4). *p < 0.05; **p < 0.01; ***p < 0.001. **b** HepG2 cells were transfected with 0.5 μg/ml of the indicated SNCG pro-Luc plasmids. Twenty-four hours later, the cells were treated with or without 5 ng/ml of TGF-β for another 24 h, followed by detection of luciferase activities. The luciferase activity in cells transfected with SNCG pro-Luc WT and treated with vehicle was set as 1. Values represent mean ± S.D. (*n* = 4). *p < 0.05; **p < 0.01; ***p < 0.001. **c** HepG2 cells were treated with or without 5 ng/ml of TGF-β for 48 h. Soluble chromatin was co-immunoprecipitated with antibody against Twist1 or histone H3 to collect Twist1- or H3-bound DNA fragments. The co-immunoprecipitated DNA was subjected to qPCR using specific primers for the SNCG promoter (SNCG-01). Values represent mean ± S.D. (*n* = 3). *p < 0.05. **d** HepG2 cells were transfected with control siRNA or siSmad3. Twenty-four hours later, the cells were treated with 5 ng/ml of TGF-β for another 48 h. Whole-cell extracts were analyzed by western blot analysis of indicated proteins. **e** HepG2 cells were transfected with control siRNA or siSmad2. Twenty-four hours later, the cells were treated with 5 ng/ml of TGF-β for another 48 h. Whole-cell extracts were analyzed by western blot analysis of indicated proteins

To further define mechanisms by which TGF-β regulates Twist1 and SNCG, we examined *SNCG* promoter activity using luciferase reporter. As shown in Fig. 4a, *SNCG* promoter activity was increased by TGF-β. Twist1 depletion repressed the promoter activity of *SNCG* and abrogated the stimulation of *SNCG* promoter activity by TGF-β (Fig. 4a). However, the activity of SNCG-E1MT promoter was significantly reduced and not affected by TGF-β (Fig. 4b). Furthermore, ChIP assays demonstrated that Twist1 binding to *SNCG* promoter was enhanced by TGF-β (Fig. 4c).

TGF-β can induce Twist1 through Smad transctiption factors^41^. Indeed, Smad2/3 knockdown compromised the induction of Twist1 by TGF-β (Fig. 4d, e). Meanwhile, Smad2/3 knockdown led to a decrease in TGF-β-induced SNCG expression, indicating that TGF-β induces SNCG through Smad-Twist1 axis (Fig. 4d, e).

### SNCG knockdown inhibits TGF-β-induced cell migration and invasion

**Fig. 5:**
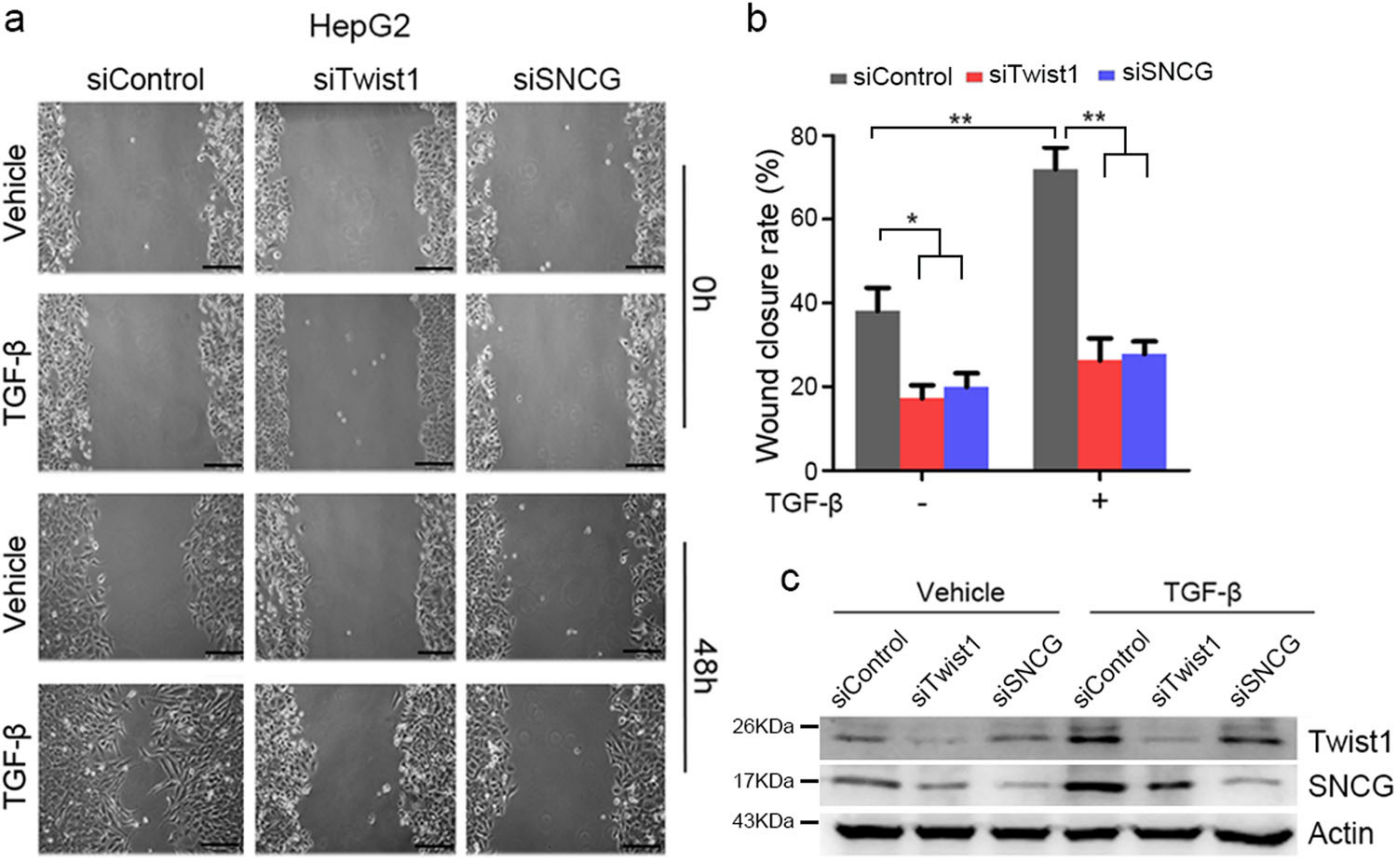
SNCG knockdown inhibits TGF-β-induced cell migration. **a** HepG2 cells were treated with 2 μg/ml mitomycin and transfected with 50 nM of indicated siRNA, and treated with or without 5 ng/ml of TGF-β, followed by wound-healing assays. Scale bar, 200 μm. **b** The wound closure rate was plotted. Values represent mean ± S.D. (*n* = 6). *p < 0.05; **p < 0.01. **c** Cell lysates were collected and subjected to western blot analysis of SNCG and Twist1 expression

TGF-β promotes cancer cells invasion and migration. To determine whether SNCG is involved in TGF-β-induced cell migration and invasion, HepG2 cells were treated with or without TGF-β and transfected with or without Twist1 and SNCG siRNA, followed by wound-healing assays. Wound area was much larger in vehicle-treated group compared with TGF-β-treated cells on day 2 after wounding, indicating that TGF-β promoted HepG2 cells migration (Fig. 5). Knockdown of Twist1 or SNCG inhibited cell migration and compromised the promotion of cell migration by TGF-β (Fig. 5). Similar effects were detected when another set of Twist1 or SNCG siRNA was transfected into HepG2 cells (Supplementary Fig. 4).

**Fig. 6:**
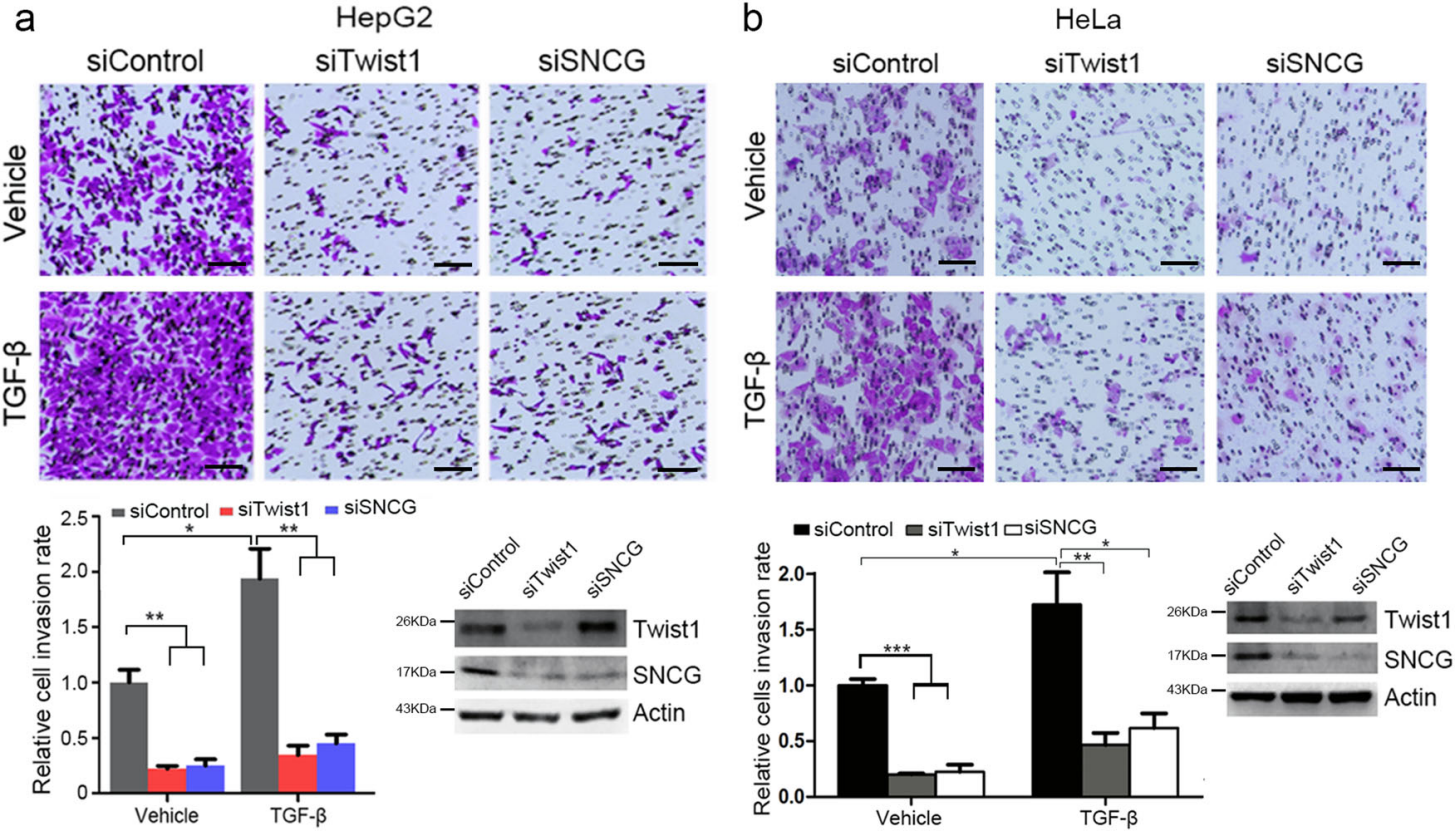
Twist1 or SNCG knockdown inhibits TGF-β-induced cell invasion. **a** HepG2 cells were transfected with 50 nM of indicated siRNA, followed by cell invasion assays in the absence or presence of TGF-β. Scale bar, 10 μm. The relative cell invasion rate was plotted. Values represent mean ± S.D. (*n* = 3). The invasion rate in siControl-transfected and TGF-β-untreated cells was set as 1. *p < 0.05; **p < 0.01. In parallel, the efficiency of Twist1 or SNCG knockdown was detected by western blotting. **b** HeLa cells were transfected with 50 nM of indicated siRNA, followed by cell invasion assays in the absence or presence of TGF-β. Scale bar, 10 μm. The relative cell invasion rate was plotted. Values represent mean ± S.D. (*n* = 3). The invasion rate in siControl-transfected and TGF-β-untreated cells was set as 1. *p < 0.05; **p < 0.01; ***p < 0.001. In parallel, the efficiency of Twist1 or SNCG knockdown was detected by western blotting

Moreover, TGF-β promoted HepG2 cells invasion, as determined by transwell assay. Knockdown of Twist1 or SNCG abrogated the promotion of cell invasion by TGF-β (Fig. 6a). Similar effects were detected in HeLa cells transfected with or without Twist1 and SNCG siRNA (Fig. 6b). These data demonstrate that, similar to Twist1, SNCG is a mediator of TGF-β-induced cell migration and invasion.

### SNCG knockdown inhibits Twist1-induced cell invasion

**Fig. 7:**
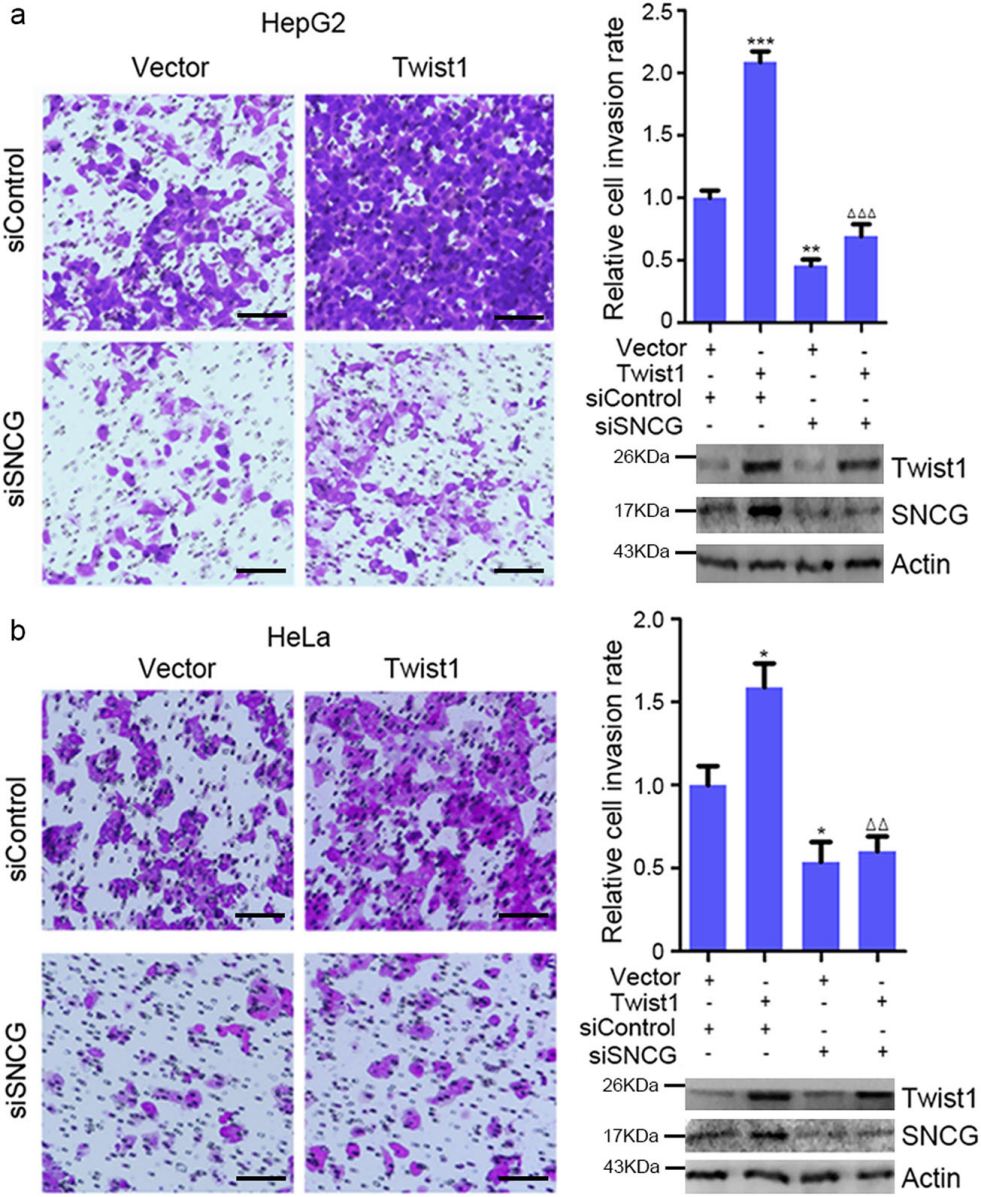
SNCG knockdown inhibits Twist1-induced cell invasion. **a** HepG2 cells were transfected with 50 nM of indicated siRNA and 1 μg/ml of Twist1 plasmid or vector, followed by cell invasion assays. Scale bar, 10 μm. The relative cell invasion rate was plotted. Values represent mean ± S.D. (*n* = 3). The invasion rate in siControl- and vector-transfected cells was set as 1. **p < 0.01; ***p < 0.001, compared with siControl; ^ΔΔΔ^*p* < 0.001, compared with siControl- and Twist1-transfected cells. In parallel, the efficiency of Twist1 overexpression or SNCG knockdown was detected by western blotting. **b** HeLa cells were transfected with 50 nM of the indicated siRNA and 1 μg/ml of the Twist1-expression plasmid or vector, followed by cell invasion assays. Scale bar, 10 μm. The relative cell invasion rate was plotted. Values represent mean ± S.D. (*n* = 3). The invasion rate in siControl- and vector-transfected cells was set as 1. *p < 0.05, compared with siControl; ^ΔΔ^*p* < 0.01, compared with siControl- and Twist1-transfected cells. In parallel, the efficiency of Twist1 overexpression or SNCG knockdown was detected by western blotting

Given that SNCG is a target of the oncogenic transcription factor Twist1, we then investigate whether SNCG acts downstream of Twist1 to promote cancer cell invasion. Indeed, overexpression of Twist1 in HepG2 cells promoted cell invasion, SNCG knockdown inhibited cell invasion and abrogated the promotion of cell invasion by Twist1 (Fig. 7a). The efficiency of Twist1 overexpression and SNCG knockdown was confirmed (Fig. 7a). Similar effects were detected in HeLa cells (Fig. 7b). These data indicate that SNCG promote cell invasion downstream of Twist1.

### SNCG knockdown inhibits the promotion of cancer metastasis by Twist1

**Fig. 8:**
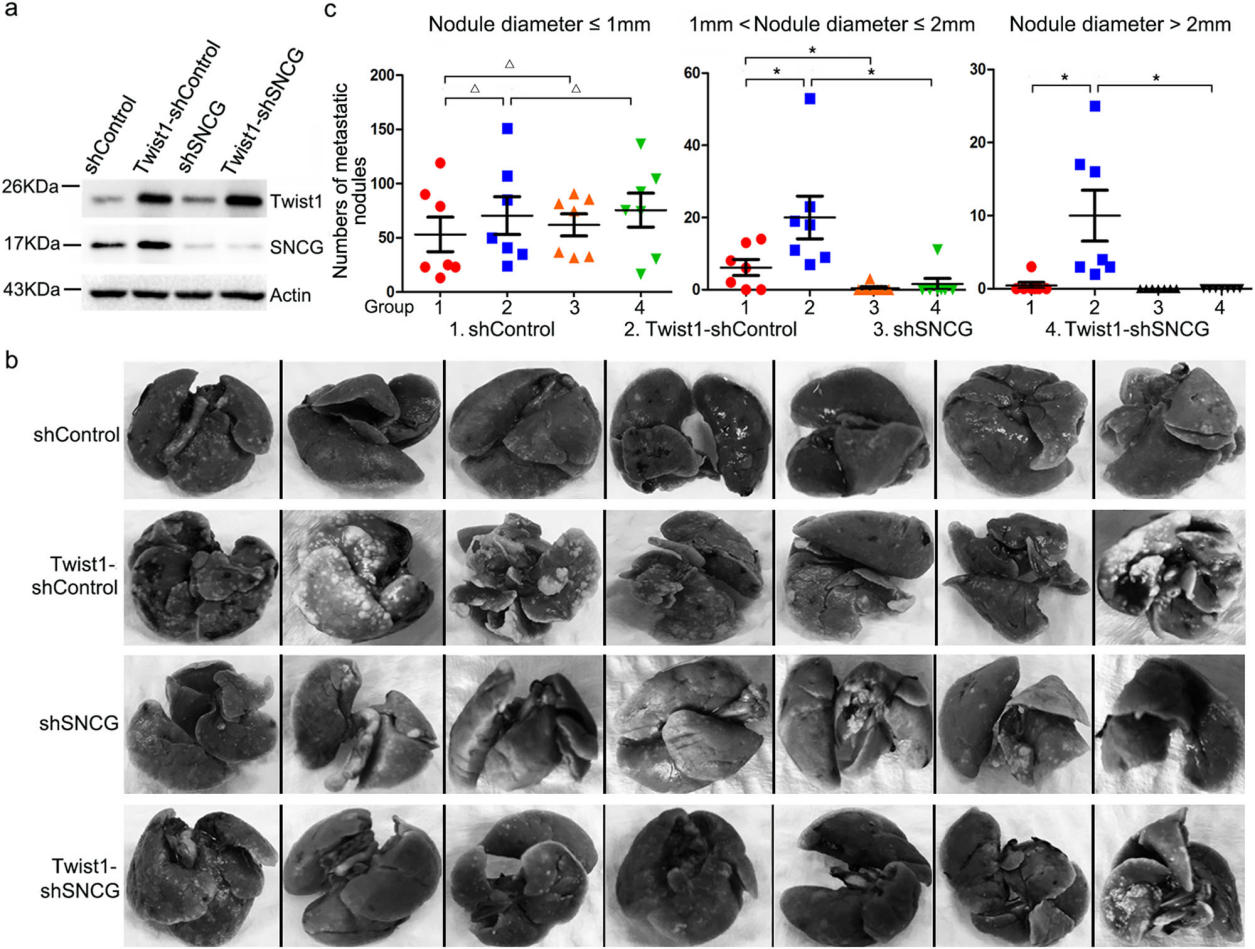
SNCG knockdown inhibits the promotion of cancer metastasis by Twist1. **a** Hela cells were transfected with lenti-shSNCG-LV3, lenti-shControl-LV3, Twist1-expression plasmid, or the negative control vector, followed by selection of stably transfected cells. Cell lysates were subjected to western blot analysis with indicated antibodies. **b** The lungs of each mouse were removed and stained in Bouin solution. Pulmonary metastatic nodules were detected. **c** Scatter dot blots show the numbers of pulmonary metastatic nodules with different size in mice inoculated with HeLa-shControl, HeLa-Twist1-shControl, HeLa-shSNCG, and HeLa-Twist1-shSNCG. The median ± SD was shown. *n* = 7 mice per group. ^Δ^*p* > 0.05. *p < 0.05

To determine the effect of SNCG on Twist1-induced cancer metastasis, HeLa cells were stably transfected with vector or Twist1-expression plasmid, followed by lentiviral transfection of shControl or shSNCG construct. Overexpression of Twist1 and knockdown of SNCG were confirmed by western blot analysis (Fig. 8a). HeLa-shControl, HeLa-shSNCG, HeLa-Twist1-shControl, and HeLa-Twist1-shSNCG cells were injected into the tail vein of nude mice. Twenty-one days later, more metastatic nodules were detected in the lungs of nude mice inoculated with HeLa-Twist1-shControl cells compared with those inoculated with HeLa-shControl, HeLa-shSNCG, or HeLa-Twist1-shSNCG cells (Fig. 8b). Further analysis demonstrated that there were significantly more lung nodules larger than 1 mm in diameter in nude mice inoculated with HeLa-Twist1-shControl cells compared with other groups (Fig. 8c). Moreover, significantly less lung nodules larger than 1 mm in diameter were detected in nude mice inoculated with HeLa-shSNCG cells compared with those inoculated with HeLa-shControl cells (Fig. 8b, c). Taken together, these data indicate that both Twist1 and SNCG promote the metastatic potential of HeLa cells, and SNCG knockdown inhibits the promotion of HeLa metastasis by Twist1.

## Discussion

TGF-β elicits cellular phenotypic changes that establish its role both as a tumor suppressor and a tumor promoter, depending on the contextual cues both in tumor cells and tumor microenvironment^37,42^. TGF-β can inhibit cell proliferation by inducing the CDK inhibitors p21Cip1 and p15Ink4b, and repressing c-myc and ID^38^. Inactivating mutations in the TGF-β pathway represents one of the mechanisms underlying evasion of the tumor-suppressive functions of TGF-β^43^. However, the core TGF-β signaling aspects are intact in many cancers such as breast cancer, melanoma, and prostate cancer, in which the tumor-suppressive arm of TGF-β is usually amputated^42^. TGF-β can also enhance the migratory and invasive properties of cancer, which represents a tumor-promoting arm of TGF-β. TGFβ1 expression in invasive cancer correlates with markers of tumor progression, such as metastasis, ECM deposition, and the infiltration of immune suppressive cells. The mechanisms underlying the switch of TGF-β from a tumor suppressor to tumor promoter may be complex. Alterations in Smad-transcription partners are important mechanisms underlying the shut-down of cytostatic programme of TGF-β^44^. In addition, p53 mutation or loss of Smad4 may contribute to oncogenic TGF-β signaling. In this study, we have demonstrated that SNCG also promotes oncogenic TGF-β signaling. Given that SNCG is usually overexpressed in late-stage cancer^11–13^, it may be another contextual cue that boosts TGF-β-driven tumor progression.

TGF-β induces the expression of SNCG through Twist1, which is a Smad target. Many extracellular stimuli affect cellular functions through induction of Twist1 expression or activation of Twist1 transcriptional activity^45,46^. The bHLH transcription factor Twist1 regulates the expression of target genes by binding to E-box promoter elements^47^. *SNCG* promoter contains multiple putative E-boxes within regions −129 to −1026 bp and −1260 to −2459 bp relative to the translation start site. Our studies demonstrate that only three of those E-boxes are important for the activity of *SNCG* promoter and the expression of SNCG. Deletion of –1260 to –2459 bp region does not affect the basal promoter activity of *SNCG*, suggesting that it is not functionally involved in *SNCG* transcription. In contrast, deletion mutations suggest that the three E-boxes within the 879 bp minimal promoter region (−879 to −1 bp) are important for the expression of *SNCG*, as deletion of this region or mutation of these E-boxes markedly reduced the basal- and TGF-β-induced promoter activity. Among the three E-boxes within the 879 bp minimal promoter region, the E-box close to the translation start site is most critical for *SNCG* transcription, as mutation of this E-box dramatically reduced basal- and TGFβ- or Twist1-induced reporter activity.

The aberrant expression of SNCG in cancer cells is likely controlled by multiple mechanisms. Previous studies demonstrate that demethylation of the CpG islands in SNCG promoter, aberrant AP1 transactivation, and IGF signaling stimulates SNCG expression^13–15^. The data presented herein clearly demonstrate that Twist1 is another positive regulator for *SNCG* transcription. Our finding that TGF-β and Twist1 positively regulate SNCG expression identifies an important signaling pathway that leads to the activation of *SNCG* gene.

Both TGF-β and Twist1 promote cancer metastasis^24^. The current study demonstrates that SNCG knockdown abrogates TGF-β- and Twist1-induced cancer cell migration and invasion, indicating that SNCG is a novel mediator of the oncogenic TGF-β-Twist1 axis. While Twist1 promotes cancer metastasis, the current study demonstrates that SNCG knockdown is able to inhibit the promotion of cancer metastasis by Twist1, suggesting that SNCG may be a pro-metastatic effector downstream of Twist1. Previous studies have demonstrated that overexpression of SNCG is correlated with cancer metastasis^11–13,16,18^. Mechanistically, SNCG may enhance cancer cell motility through activation of RHO family small-GTPases and ERK^48^. SNCG may promote cancer progression downstream of Smad-Twist1 axis in TGF-β signaling. Elucidation of the molecular mechanism underlying the aberrant expression of SNCG in cancer may provide insights into the development of therapeutic approaches to antagonize SNCG expression and to inhibit cancer progression.

## Materials and methods

### Cell culture

Hepatoma cancer cell lines HepG2, cervical cancer cell lines HeLa were obtained from Cell Lines Bank, Chinese Academy of Science (Shanghai, China). The cells were maintained in DMEM supplemented with 10% new born calf serum (Thermo Fisher Scientific, Waltham, MA, USA). The cells were incubated at 37 °C in a humidified atmosphere of 5% CO_2_.

### Reagents and antibodies

TGF-β was purchased from PeproTech (USA). The antibodies used were as follows: anti-Twist1 was purchased from Abcam (Cambridge, UK); anti-SNCG and anti-β-actin were purchased from Santa Cruz Biotechnology (Santa Cruz, CA, USA); anti-Smad2 and anti-Smad3 were purchased from ABclonal (Wuhan, China); anti-phospho-Smad2 (S245/250/255) and anti-phospho-Smad3 (S423/425) were from Cell Signaling Technology (Danvers, MA, USA). All siRNAs were custom-synthesized products of Ribobio Co., Ltd. (Guangzhou, China). The siR-Ribo negative control (siControl) was used for all siRNA experiments. The target sequences for Twist1 knockdown are as follows: 5′-GGUACAUCGACUUCCUCUA-3′ for siTwist1#1, and 5′-UUGAGGGUCUGAAUCUUGCUCAGCU-3′ for siTwist1#2. The sequence of siRNA targeting the 3′-untranslated region of Twist1 gene (siTwist1#3) is 5′-CACCTCTGCATTCTGATAGAA-3′. The two target sequences for SNCG knockdown are as follows: 5′-GCAGCTGAGAAGACCAAGG-3′ for siSNCG#1 and 5′-GGAGAATGTTGTACAGAGC-3′ for siSNCG#2. Target sequences for Smad3 are 5′-GGAGAAAUGGUGCGAGAAG-3′.

### Plasmids construction

Plasmid for Twist1 (pBABE-puro-mTwist1#1783) was purchased from Addgene (Cambridge, MA, USA). The complementary DNAs (cDNAs) were subcloned into pcDNA3.1(+) (Invitrogen). PGL3 luciferase reporter vectors contain four plasmids: PGL3-Basic vector, PGL3-Enhancer vector, PGL3-Promoter vector, and PGL3-Control vector, were purchased from Promega (Madison, USA). The SNCG promoter (−1/−2486 bp) reporter (SNCG pro-Luc WT) was constructed by our own laboratory. The cDNAs encoding SNCG promoter region were amplified by PCR from HepG2 cells by using following primer pairs: 5′-CGGGGTACCGGGCAGGCGGGAATGAGGTTTCTC-3′ (forward) and 5′-CCCAA GCTTGGTGGGTGTGCAGGGTTGTGCTG-3′ (reverse), and then subcloned into PGL3-Basic Vector (Promega). Various derivatives of SNCG promoter plasmids (SNCG pro-Luc −129/−1026del; SNCG pro-Luc −1260/−2459del) were amplified by PCR from SNCG pro-Luc WT plasmid using following primer pairs: 5′-AGCTCTGCAGATCAGAGAGGCTAGTACT-3′ (forward) and 5′-ATGGAGAAACCTCATTCCCGCCTGCCC-3′ (reverse) for SNCG pro-Luc −1260/−2459del.; 5′-TTTCATCGGCGTCAATAGGAGGCATC-3′ (forward) and 5′-GCCACTTCCTCTCTTTGTTGTCCTCG-3′ (reverse) for SNCG pro-Luc −129/−1026del. Mutated SNCG promoter reporter (SNCG pro-Luc E1MT; SNCG pro-Luc E2MT; SNCG pro-Luc E3MT) were constructed by GENEWIZ (Suzhou, China). Lentiviral vectors for short hairpin RNA against SNCG (shSNCG) and shControl were purchased from GenePharma (Shanghai, China).

### Transfection

For transient transfection of plasmids, cells were transfected using Lipofectamine 2000 (Thermo Fisher Scientific, Waltham, MA, USA). For transfection of siRNA, proliferating cells in six-well plates were incubated with 50 nM siRNA in 2 ml of OPTI-MEM^®^ I Reduced Serum Medium (Life Technologies, Carlabad, CA, USA) containing Lipofectamine^®^ RNAiMAX (Thermo Fisher Scientific). Lentiviral packaging of recombinant vectors of lenti-shSNCG-LV3 or lenti-shControl-LV3 targeting SNCG gene sequences or a nonsense sequences was produced in 293T cells using a standard protocol. Forty-eight hours after transfecting HeLa cells with these lentivirus, the cells were treated with 2 μg/ml of puromycin. Two weeks later, the puromycin resistant clones were selected and expanded in 1 μg/ml puromycin-containing growth medium. Knockdown of SNCG was confirmed by western blot. For stable tranfection of Twist1 or the negative control vector, shControl- and shSNCG-transfected HeLa cells were transfected with pcDNA3.1-Twsit1 or the negative control pcDNA3.1 using Lipofectamine 2000. Forty-eight hours later, the cells were treated with 800 μg/ml of G4l8 and 1 μg/ml puromycin. Two weeks later, the G4l8-resistant clones were selected and expanded in 400 μg/ml G418 and 1 μg/ml puromycin-containing growth medium.

### Western blot analysis

Whole-cell extracts were obtained by lysis of cells in an appropriate volume of ice-cold radioimmunoprecipitation assay (RIPA) buffer (1% Triton X-100, 40 mM HEPES, pH 7.5, 120 mM NaCl, 1 mM EDTA, 10 mM pyrophosphate, 10 mM glycerophosphate, 50 mM NaF, 0.5 mM orthovanadate) containing protease inhibitors PMSF, aprotinin, and phosSTOP (Roche, Indianapolis, IN, USA). Cell lysates were incubated on ice for 35 min and then centrifuged for 15 min at 13,000×*g* to remove debris. Aliquots of proteins were boiled in 1× loading buffer for 10 min, samples containing 30 µg of total proteins were resolved by SDS-PAGE, and proteins transferred to PVDF membrane (Millipore Corporation, Billerica, MA, USA). Membranes were incubated with primary antibodies overnight at 4 °C and appropriate HRP-secondary antibodies for 1 h at room temperature. Detection was performed with chemiluminescent agents. Images were gathered by Alpha Innotech’s FluorChem imaging system.

### Immunoprecipitation

Cells were lysed in the RIPA buffer containing protease inhibitors. Cell lysates were incubated on ice for 30 min and then centrifuged for 20 min at 13,000 r.p.m. to remove debris. The clarified cell lysates (1 mg total proteins) were used for immunoprecipitation with primary antibodies or normal immunoglobulin G at 4 °C overnight. Then the supernatants were incubated with 30 μl protein G agarose beads for 2 h at 4 °C. After washing in lysis buffer five times, bound proteins were analyzed by western blotting.

### Quantitative reverse transcription PCR analysis

Total RNA were extracted from cultured cells using Trizol reagent (Thermo Fisher Scientific) according to the manufacturer’s protocol. First strand cDNAs were synthesized using the MMLV reverse transcriptase and oligo(dT) primers. GAPDH was amplified by real-time PCR using the SYBR^®^ Select Master Mix (Thermo Fisher Scientific). The primer sequences for human *Twist1* were 5′-GTCCGCAGTCTTACGAGGAG-3′ (forward) and 5′-TGGAGGACCTGGTAGAGGAA-3′ (reverse). The primer sequences for human *SNCG* were 5′-ATGCGGCTGCCCACGCTCCT-3′ (forward) and 5′-GTCTTGGCTCCCACATACAT-3′ (reverse). The primer sequences for *GAPDH* were 5′-AATCGCATCATCATAACCTG-3′ (forward) and 5′-CATCCTGCCCATCATACTC-3′ (reverse). Relative quantification with the comparative threshold cycle (Ct) was done using the Ct method. The amount of *Twist1* and *SNCG*, normalized to the endogenous reference gene (*GAPDH*) is given by 2−ΔCt, where ΔCt is Ct (*Twist1* or *SNCG*) − Ct (*GAPDH*).

### Luciferase reporter assay

Cells were seeded in 24-well plates, then transfected with the indicated reporter plasmid, expression plasmid, siRNA, and pRL-TK as an internal control. The luciferase activities were measured using a dual-luciferase reporter assay system (Promega, BioSciences, San Luis Obispo, CA, USA) and a luminometer (Thermo Fisher, Waltham, USA). The Firefly luciferase activities were normalized by the corresponding Renilla luciferase activities.

### Chromatin immunoprecipitation assay

ChIP assays were performed with the ChIP system according to the manufacturer’s instructions (Millipore Corporation, Billerica, MA, USA). Briefly, cells were fixed with formaldehyde for 10 min at room temperature. After washing twice with 1× cold PBS containing proteinase inhibitor cocktail II, the cells were scraped and lysed with cell lysis buffer and nuclear lysis buffer, and then the DNA was broken into pieces 200–1000 bp in length by sonication. Soluble chromatin was co-immunoprecipitated with antibody against Twist1, H3, or IgG at 4 °C overnight. The de-crosslinked DNA samples were subjected to PCR. The primer sequences for SNCG-01 were 5′-TGGAGGGATGAACTGAGATA-3′ (forward) and 5′-ATCCTGCTGATGAGGTGTT-3′ (reverse). The primer sequences for SNCG-02 were 5′-AGGGACTCTGGGAATGTGG-3′ (forward) and 5′-TGCTGAAGGTGCTGAGGAG-3′ (reverse). The primer sequences for SNCG-03 were 5′-AGGATGCTGGTGGGTAGCT-3′ (forward) and 5′-CAACCTGTCCCTCATTCATTTA-3′ (reverse). The primer sequences for SNCG-04 were 5′-TGGAGGAAGGTGAGGCTGAA-3′ (forward) and 5′-CTCCTATTGACGCCGATGAA-3′ (reverse).

### Cell migration and invasion assay

Cell migration was determined by wound-healing assay. Cells were seeded in six-well plates at 50,000 cells per well. Twenty-four hours after the plating, the cells were treated with 2 μg/ml mitomycin C to inhibit cell proliferation. The indicated siRNA or plasmid was transfected into the cells. Twenty-four hours after transfection, a wound was made in the monolayer by pressing a pipette tip down on the plate. The debris was removed by washing the monolayer twice with serum-free media, and the cells were cultured in serum-free media with or without 5 ng/ml of TGF-β for an additional 48 or 72 h. Cell migration was recorded in six different microscopic fields. The area of scratch was recorded by taking images under a phase-contrast microscope and the gap width was measured by NIS-Elements F3.0 imaging system (Bio-Rad Laboratories, Hercules, CA, USA). The percentage of wound healing was calculated by the equation: (percentage of wound healing) = average of [(gap area: 0 h)—(gap area: 48 or 72 h)/(gap area: 0 h)].

Cell invasion was detected using transwell chambers (Millipore Corporation, Billerica, MA, USA) with Matrigel (BD Biosciences) covered. Each chambers were uniformly covered with 60 ml Matrigel diluted with DMEM to a certain percentage and incubated at 37 °C for 1 h. HepG2 cells (8 × 10^4^) or HeLa cells (1 × 10^5^) transfected with the indicated siRNA or plasmid were applied in the upper compartment with 500 μl of serum-free medium, and the lower compartment was filled in 500 μl of DMEM with or without 10 ng/ml of TGF-β. After 24–48 h of incubation at 37 °C (HepG2, 24–36 h; HeLa, 36–48 h), noninvaded cells on the upper surface of the filter were removed carefully with a cotton swab, and cells were fixed with 100% methanol for 2 min. Invaded cells on the lower side of the filter were stained with 0.5% crystal violet for 20 min, and images were captured using a fluorescence microscope. The invaded cells’ area on the lower side of the filter was evaluated by Image Pro Plus 6.0. software.

### In vivo cancer metastasis assay

Five-week-old female athymic nu/nu mice were purchased from Slaccas Company (Shanghai, China) and randomly divided into four groups of 7 mice each. 2 × 10^6^ HeLa-shControl, HeLa-shSNCG, HeLa-Twist1-shControl, and HeLa-Twist1-shSNCG cells were resuspended in 0.2 ml of PBS and injected into the tail vein of nude mice, respectively. Mice were killed at 21 days post injection. The lungs of each mouse were removed. To quantify lung metastasis, lungs were stained in Bouin solution for 48 h, followed by rinsing with 70% (v/v) ethanol. The size of metastatic nodules was measured under anatomy microscope. Animal handling and procedures were approved and performed according to the requirements of the Institutional Animal Care and Use Committee of West China Hospital.

### Statistical analysis

One-way analysis of variance with post hoc tests was used in statistical analysis of messenger RNA expression, luciferase activity, cell migration, and invasion. For statistical analysis of lung metastasis, *p* values were calculated by unpaired Student’s *t*-tests. All statistical tests were two-tailed. Differences were considered statistically significant if *p* < 0.05.

## Electronic supplementary material

Supplementary figures and legends
