# Supplementary material for: Gamma synuclein is a novel Twist1 target that promotes TGF-β-induced cancer cell migration and invasion
Source: Cell Death Dis. 2018 May 24;9(6):625. doi: 10.1038/s41419-018-0657-z (PMC5967338; doi:10.1038/s41419-018-0657-z)
Supplement: Supplementary file 1 — Supplementary figures and legends [file 41419_2018_657_MOESM1_ESM.pdf]

Gamma synuclein is a novel Twist1 target that promotes TGF-β-induced cancer cell migration and invasion

Supplementary Information

SNCG promoter -1/-2486bp (https://www.ncbi.nlm.nih.gov/gene/6623)

E-box caXXtg

-2486 gggcaggcgggaatgaggtttctccatggc**caactg**ctcagccactgttccccacgccactgccaccagaccctcactaa

-2406 ggggcctcttgcccctccctggtcaaatctgttttcaggaaagcacccaacaaacctgcaactcagaagc**caaatg**agac

-2326 ctatcccaggcaggtccgc tctgcgatggtggctctcatacaccgcacagaagtgtaagttggccccccctcactccc

-2247 taaatccatgtagatgcccttagccaaacaggtctggtgtgtgggggcaggagggtggaggtaggag**catctg**gggctg

-2167 gggctggctc**caagtg**ctgcagagagttcactatgagctctgaagtcaaactcagatcctgctccttagctgtgtggcct

-2087 tgggcaagcccctcagcctctctgagcctcaggctcctcctctgtaagtggggctgttggagggatgaactgagata**cac**

-2007 **gtg**gcaaggtgcctggccctgagactgggggccccgtaaacag**cacctg**ctgctctgactgagggg**cagttg**gagatgag

-1927 gggggacttacaggcccaacacctcatcagcaggatgggccctgg ctctgtacacatagaatgttggaaagctatgttc

-1848 tgggtc **cagttg**caagaccatcctgcc**cacttg**ctgggtcgctgaggcagagcgccctggactgtgctctggagagcag

-1769 ggggccgtctctgctcactgctgggcagtcagagtcaggtccccagggaactcagatcaaaatctcagggcagccttgct

-1689 gggtctcagctttcagaattgatttctttggaagaacaggaggcatagccaggc**cagctg**gcaatggcttcagccacagt

-1609 cccccagtgccccatgctgggtgattgatggtaggagctgtgcctccctgccagcccccaagccccattccctgccatg

-1529 gctccgacagggaactccctgcaattcttaggtggatcctgaaacacccagtccagactacttttcagcctca**cagatg**

-1449 gcccagagatagatactattagcccatttca**cagatg**ggaccccagtgaggtctagagaggctctgctctagctgagctg

-1369 ggactcagatcctactccatgacctctgccaaggtccaggccctcttaccgtcctacggggtccttgccggtgtcctcag

-1289 cctctgccttccagaccc**caggtg**tcctggagctctgcagatcagagaggctagtactggaagcctgggcccatgccccc

-1209 agcagcccccagcccagggggcccccaaggctgaacagcaagctcaggatcatcttggtggtggggcaggctcagctcac

-1129 actcagccttggcaagtagctccagaaactgctagtgacgttgtcttcaagttaaatctcaggaggaaaagaaaatacga

-1049 ggacaacaaagagaggaagtggcctgggccggcctacccggtgggtcttgtcctgccccccaactaccctggctggcccc

-969 acaggggccgccaaccacacaagccagttcctgtccctgaggacttggctcagggactctgggaatgtggtagacatggg

-889 gtggccccac**caaatg**catccttatgggaacctgctccctgggagccatgaaaagagcgtggacttcgaggtggggccac

-809 aggaagtggtcaggtccatctcaggggacctgctgcccatccacactgctggccaggaaatgggggcaattcatgcctc

-729 ctcagcaccttcagcactgggcggctcaaagaagggaagggactattctggggtcacacagcatgcagccagaggccaag

-649 gcatgaggaagtccttcatttccccacccccacccacctcagatcctccaaccggtttcatggcagcccagggtccagcg

-569 gcatccaggatgctggtgggtagctgcacagcccaggccgcgggaggttggctgctctcacctaacaggcctatgtggcc

-489 ctgacccctacctaggaagctggggacaatggccaaggcgcctcccctctctgtgcctgtctgtc**caggtg**cagcataga

-409 cacagcaccccctggggccaagagcacccagccagggctgcccccatgggtgggcagggcagtaaatgaatgagggacagg

-329 ttgggaggtggccagcccccctccagcccatggagggcacggggcaggagagctgggctgagccagcaggagcccagggag

-249 cctggtctctgccttcctatcctggaggaaggtgaggctgaacctccttccctccctccctccctccccgcccccactgc

-169 acgcagggctggctgggctc**cagctg**gcctccgcatcaatatttcatcggcgtcaataggaggcatcggggacagccgct

-89 gcggcagcactcgagccagctcaagcccgcagctcgcagggagatccagccctgcagtccgtcctgcagcacaaccctgc

-9 acacccacc -1

Suppl. Fig. 1

Supplemeantary Figure 1: E-boxes within SNCG promoter.

The sequence of SNCG promoter (-1/-2486bp) was from NCBI. There are 16 E-boxes (caXXtg) in the indicated region of SNCG promoter (-1/-2486).

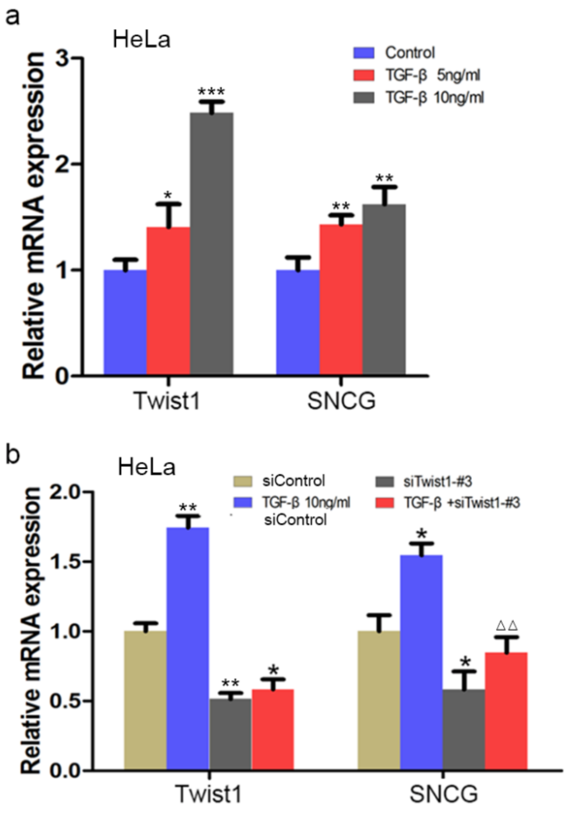

Suppl. Fig.2

Supplemeantary Figure 2: Twist1 knockdown inhibits TGF-β-induced SNCG transcription in HeLa cells.

(a) HeLa cells were treated with TGF-β for 24h at the indicated doses, followed by qRT-PCR analysis of *Twist1* and *SNCG* transcription. The levels of transcripts in vehicle-treated cells was set as 1. Values represent mean ± S.D. (n = 3). *, $p < 0.05$; **, $p < 0.01$; ***, $p < 0.001$, compared with vehicle-treated cells. **(b)** HeLa cells were transfected with siControl or siTwist1#3. 24 hours later, the cells were treated with 10 ng/ml of TGF-β for another 24h, followed by qRT-PCR analysis of *Twist1* and *SNCG*, transcription. The levels of transcripts in vehicle-treated and siControl-transfected cells was set as 1. Values represent mean ± S.D. (n = 3). *, $p < 0.05$; **, $p < 0.01$, compared with siControl; △△, $p < 0.01$, compared with TGF-β-treated and siControl-transfected cells.

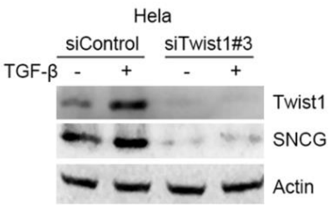

Supplemeantary Figure 3: TGF-β induces SNCG expression through Twist1.

HeLa cells were transfected with siControl or siTwist1#3. 24 hours later, the cells were treated with 10 ng/ml of TGF-β for another 48h, followed by western blot analysis of Twist1 and SNCG expression.

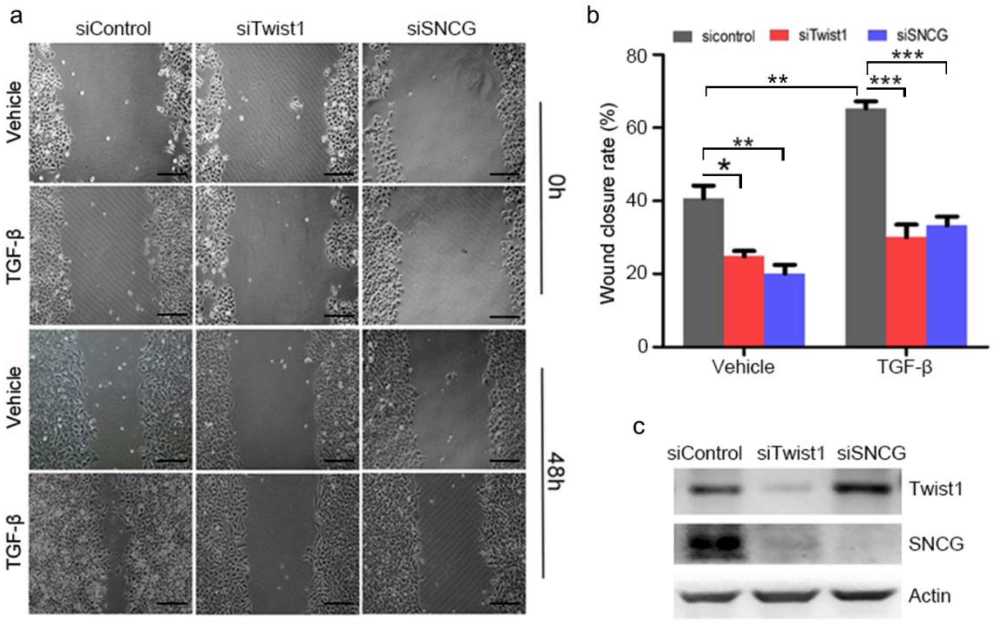

Suppl. Fig. 4

Supplemeantary Figure 4: SNCG knockdown inhibits TGF-β-induced cell migration

(a) HepG2 cells were treated with 2 μg/ml mitomycin and transfected with 50 nM of siControl or siSNCG#2, and treated with or without 5 ng/mL of TGF-β, followed by wound-healing assays. *Scale bar*, 200 μm.. **(b)** The wound closure rate was plotted. Values represent mean ± S.D. (n = 6). *, $p < 0.05$; **, $p < 0.01$; ***, $p < 0.001$. **(c)** The efficiency of SNCG and Twist1 knockdown in TGF-β-treated cells was detected by western blotting.
